# Artmaking Across Media: An Expressive Therapies Continuum Perspective on Stress Reduction Through Artmaking with Digital vs. Traditional Materials

**DOI:** 10.3390/bs16050645

**Published:** 2026-04-25

**Authors:** Or Chen Halbrecht-Shaked, Johanna Czamanski-Cohen, Aviv Sion

**Affiliations:** 1 School of Creative Arts Therapies, Faculty of Social Welfare and Health Sciences, University of Haifa, Abba Khoushy Ave 199, Haifa 3498838, Israel; halbrechtor@gmail.com (O.C.H.-S.);; 2 Emili Sagol Creative Arts Therapies Research Center, Faculty of Social Welfare and Health Sciences, University of Haifa, Abba Khoushy Ave 199, Haifa 3498838, Israel

**Keywords:** art therapy, Expressive Therapies Continuum, digital art

## Abstract

Artmaking is recognized as an effective means of supporting emotional regulation and reducing stress, yet little empirical work has directly compared the psychological and physiological effects of digital versus traditional art materials. Guided by the Expressive Therapies Continuum (ETC), this study examined whether drawing with oil pastels on paper or drawing on a digital tablet differentially influenced emotional state, physiological stress, and subjective creative experience following a validated group stress induction. Forty-eight healthy adult women were randomly assigned to create art for 45 min using either oil pastels or a tablet with a digital stylus. Measures included state anxiety, salivary cortisol, emotional valence, arousal, dominance, flow experience, and artmaking experience. Both modalities produced significant reductions in state anxiety, with no differences between groups. Emotional responses also changed significantly from pre- to post-artmaking, again without between-group differences. Cortisol levels did not significantly decrease in either condition, and no differences emerged across flow dimensions or artmaking experience scales. These findings indicate that tablet-based and traditional oil pastel drawing generate comparable emotional and experiential benefits following acute stress. Interpreted through the ETC, results suggest that therapeutic mechanisms of artmaking may be activated across a wider range of media than previously assumed. Digital tools appear capable of facilitating sensory–affective and integrative processes often attributed to traditional materials, thereby supporting their integration into trauma-informed practice.

## 1. Introduction

Visual art creation is associated with changes in emotional and physiological indicators. The ETC provides a theoretical framework to help us understand how artmaking provides opportunities for cognitive, emotional, and sensorimotor processing. Laboratory studies of artmaking can help determine whether it is the interaction and physical contact with the art materials themselves or other factors that cause psychological and physiological changes following artmaking.

Each art material has unique characteristics based on its level of fluidity, texture, and structure, all of which point to different therapeutic potentials. Kagin and Lusebrink (1978) developed the Expressive Therapies Continuum (ETC), which describes the interdependence between art materials and the client’s physical state. According to this model, progression through art materials corresponds to the client’s psychological and physiological readiness. Materials with high structure tend to evoke cognitive expression, whereas fluid materials tend to evoke emotional experience. Client preferences for specific materials may also reflect states of sensory over-processing or information-processing blocking.

The model describes three bipolar levels: kinesthetic/sensory, perceptual/affective, cognitive/symbolic, and an additional creativity level, which may appear at any level or integrate between levels. Furthermore, the ETC model suggests that controlled media (markers, pencils) strengthen kinesthetic, perceptual, and cognitive components. Fluid media (gouache, finger paints) strengthen sensory, emotional, and symbolic components. As a result, researchers have examined the connection between the fluidity of materials and emotional/physiological responses. A quasi-experimental study among 50 adults examined responses to drawing with pencil, oil pastels, and gouache. It found that oil pastels had a uniquely strong effect on heart rate variability, gouache had a moderate effect, and pencil had no effect (Haiblum-Itskovitch et al., 2018).

In recent years, the use of tablets as a tool for stress reduction in art therapy has increased. Despite this growing use, few studies have examined its implications in therapeutic contexts. One main concern regarding digital media relates to whether it can satisfy the sensory needs that traditional art materials provide. Therefore, it is important to examine the emotional and physiological effects of creating with digital tools compared to traditional materials. Consequently, the purpose of the current study is to examine stress reduction following artmaking, using both emotional and physiological measures (cortisol levels), while comparing drawing on a tablet with drawing using traditional materials.

When a person is exposed to stress-inducing stimuli, various behavioral and physiological responses may arise, depending on how the individual perceives the internal or external event as threatening. Cortisol is the main hormone in the family of glucocorticoid hormones and a physiological marker of stress.

With a small saliva sample, one can non-invasively measure cortisol levels. A study that examined cortisol levels in saliva following free-artmaking, as well as the creators’ subjective responses, found that after approximately 45 min there was a significant decrease in cortisol levels in saliva (Kaimal et al., 2016).

In the field of creative arts therapies, there is an increasing demand for empirical evidence supporting clinical practice. Accordingly, in recent years, more art therapists have called for research examining the effectiveness (or lack of effectiveness) of specific art therapy methods (Kimport & Robbins, 2012). Likewise, there has been a rise in studies exploring the emotional effects and subjective experience associated with creating using traditional art materials, while very few studies have examined the physiological effects of these materials on clients. Research indicates a connection between visual artmaking and changes in both emotional and physiological measures (Haiblum-Itskovitch et al., 2018; Kaimal et al., 2016).

Despite these findings, it is still unclear which specific elements of artmaking lead to these physiological changes, whether it is the interaction with the materials, the sensory engagement, or other factors (Kaimal et al., 2016; Haiblum-Itskovitch et al., 2018).

Art materials serve as a central tool in art therapy (Malchiodi, 2012). They also hold a symbolic meaning, enabling clients to express ideas, thoughts, and emotions (Moon, 2010). Therefore, the interaction between the client and the art materials is of great importance (Pénzes et al., 2016). Through this interaction, the art therapist can understand the client’s flexibility, motivation, and rationalization by observing and analyzing behavior through concepts such as rhythm, space, order, control, development, movement, pressure, repetitiveness, and the giving of form (Pénzes et al., 2014).

In addition, the therapist must adapt the art materials offered in therapy according to the progress of the therapeutic relationship (Hinz, 2009). It is suggested, by some that the therapist first offer controlled and rigid materials (e.g., pencils, markers). These materials limit emotional expression but foster a sense of safety and comfort without fear of emotional overwhelm. As the therapeutic relationship strengthens, a broader range of more fluid materials that allow for richer emotional expression should be introduced, for example, oil pastels (Snir & Regev, 2013). Digital media have increasingly entered the therapy room alongside traditional art materials. However, very few studies have explored the implications of this technological development or its meaning within the therapeutic process (Carlton, 2014). One of the most widespread digital tools in recent years is the tablet (Orr, 2012).

As electronic platforms have become more common, their therapeutic and creative potential has grown (Orr, 2012; Garner, 2018). The use of digital formats in art therapy has expanded substantially as art therapists increasingly recognize the relevance of digital media within clients’ everyday lives and the broader visual culture they inhabit (Orr, 2012). Digital tools offer distinct advantages over traditional art materials, including portability, affordability, reduced mess, and the capacity for easy editing, saving, and revisiting artwork, thereby accommodating a wide range of developmental, physical, and emotional needs (Choe, 2014). Empirical and clinical literature further indicate that digital media can enhance therapeutic engagement, particularly among youth who are fluent in digital environments, by offering familiar interfaces and interactive features such as layering, undo/redo capabilities, multimedia integration, and process recording (Choe, 2014; Orr, 2012). Case studies demonstrate that such tools support emotional regulation, narrative restructuring, and reflective functioning; for example, the use of iPad art apps enabled an 11-year-old client to develop greater distress tolerance, cognitive flexibility, and empathic understanding, while digital journaling facilitated a 16-year-old client’s mood monitoring, insight development, and self-regulated coping within the context of bipolar disorder (Choe, 2017). Central to the effective integration of these technologies is the concept of digital literacy, defined as the ability to access, evaluate, synthesize, and ethically manage digital resources, a competency increasingly essential for contemporary art therapists operating within technologically saturated clinical and cultural landscapes. Despite concerns regarding loss of tactile experience, potential dependency on devices, or risks to privacy and confidentiality, research suggests that these challenges can be mitigated through informed consent, technological safeguards, and clinically attuned decision-making about when and for whom digital media are indicated (Choe, 2014).

A wide variety of artmaking applications can now be downloaded to these devices and used in a personalized way. Another advantage of digital platforms is that the applications are designed in an intuitive visual format, featuring icons that represent different art materials (stickers, stamps, pencils, chalk, etc.), making them easy and accessible to use (Choe, 2014; McLeod, 1999). Collectively, the emerging scholarship suggests that digital formats, when implemented with clinical sensitivity and ethical rigor, not only expand expressive possibilities but also strengthen art therapy’s relevance within contemporary digital culture. Despite this technological progress, several questions arise regarding the meaning and implications of introducing digital media into the therapy room alongside traditional art materials. One source of uncertainty concerns the therapeutic alliance, whether digital media might interfere with the development of the relationship between the therapist and the client. Another question relates to the sensory experience associated with classical art materials, and whether the absence of this sensory component in digital tools limits the client’s ability to express emotions fully. These questions are further intertwined with preexisting biases against digital media, particularly within therapeutic contexts (Carlton, 2014).

Additionally, artwork created through digital means is often valued less than work created with traditional art materials, particularly due to the perceived absence of direct tactile engagement with materials (Orr, 2006). However, it is increasingly recognized that digital platforms may serve as a meaningful material alternative because many children and adolescents perceive digital media as a comfortable and natural space for expression and communication. Digital devices can also be easily sanitized, making them useful for work with vulnerable or immunocompromised populations, such as oncology patients (Nainis, 2008).

To date, very few studies have directly compared the processes and artistic products created with traditional materials versus those created with digital tools (Carr & Vandiver, 2003; Backos et al., 2014). Therefore, research in this area is essential for developing professional discourse around digital tools and deepening our understanding of their therapeutic implications (Carlton, 2014). A quasi-experimental study of 35 children and adolescents (26.5% on the autism spectrum) examined attitudes toward digital media among children, adolescents, and parents and compared drawings made with colored pencils vs. drawings made on tablets. The study found no difference in the participants’ experience of creating with the two media, nor in the time spent drawing with either method. Drawings made on the tablet were more colorful and covered more visual space. However, tablet drawings reflected a lower developmental level than drawings made on paper (Thomas, 2017).

This study contributes to the existing body of knowledge and supports clinical assumptions regarding the use of tablets within the art therapy setting. In addition, it helps dispel preconceived notions about the use of digital media in art therapy. Moreover, this study offers another layer of understanding regarding the significance of direct tactile engagement with materials during the creative process and its influence on physiological and emotional changes. The importance of this research also lies in the fact that it is one of the first studies to examine physiological changes occurring after artmaking using both traditional materials and tablet-based materials. Therefore, this study encourages re-evaluation of the use of traditional art materials and raises awareness of the thoughtful, individualized selection of creative materials according to the client’s emotional state and needs.

The purpose of the research is to compare the effects of two modes of art making: drawing with oil pastels on paper and drawing on a tablet using an electronic stylus. The comparison focuses on stress, emotional state, and physiological measures, specifically cortisol levels in saliva. The research question is: Are there emotional and physiological differences when creating art on a tablet compared to creating art with traditional art materials? We hypothesize that cortisol levels and state anxiety decrease following artmaking with both the tablet and traditional art materials (H1). Participants report a greater sense of flow and a stronger emotional response when drawing with traditional art materials compared to drawing on a tablet (H2).

## 2. Materials and Methods

To examine the research hypotheses, a controlled comparative experiment was conducted in which participants were randomly assigned to draw on a tablet or with oil pastels after a stress induction protocol. Psychological and physiological stress measures were collected before and after the induction.

### 2.1. Participants

The experiment was conducted with 48 healthy women between the ages of 19 and 55, with a mean age of 22.85 (±5.31). Participants were recruited from the community, social media, and the University of Haifa. Participants from the university received course credit in an introductory psychology class. Inclusion criteria: age 18 or older, no cardiac pathology or recent heart attack, not taking anticholinergic medication regularly, and sufficient proficiency in Hebrew.

### 2.2. Procedure

Forty-eight women arrived at the laboratory at 16:00 and were randomly assigned to draw with oil pastels or with a digital stylus on a tablet. A group stress induction was conducted using the Sing-a-Song Stress Test Group (SSST-G), with groups of 5 to 10 participants (Brouwer & Hogervorst, 2014). The rationale for inducing stress in this protocol, rather than comparing drawing with both materials without induction, was that we were interested in the role of drawing specifically for stress reduction. We were concerned that without an induction of stress, it would be more difficult to measure an effect. Drawing on the tablet (iPad Pro 12″) was done using the ArtRage app and a digital pen, using only the oil pastel feature of the application. Those drawing with traditional materials viewed the SSST-G presentation on an iPad 5th Generation.

**Figure 1:**
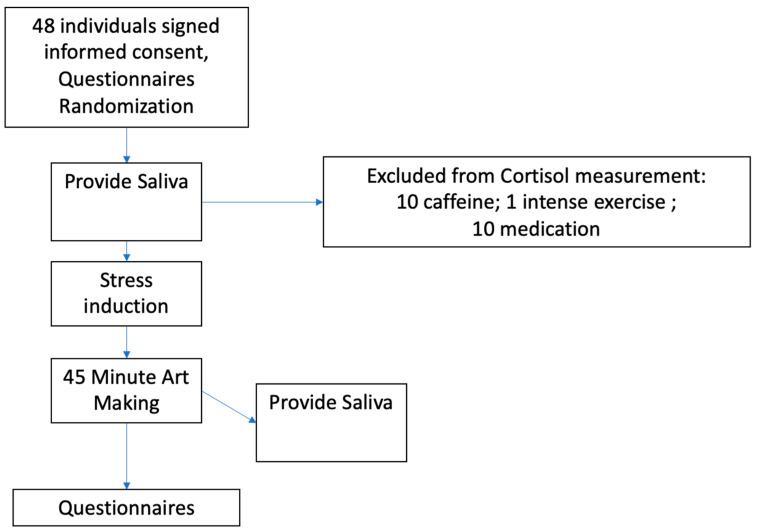
Flow chart of the experiment.

Saliva was collected for the measurement of cortisol levels before the stress induction and after 45 min of drawing. Psychological questionnaires were administered at three time points as shown in Figure 1. Participants were tested in the late afternoon (16:00–18:00) to ensure optimal cortisol measurement. They received an explanation of the procedure, signed informed consent, removed their watches, and turned off their phones. Twenty-one participants were excluded from cortisol analyses as they did not follow the protocol of no caffeine, exercise, or anticholinergic medications before the experiment; thus, the results of the cortisol reflect only 27 participants.

During SSST-G, participants sat at a table and silently read neutral sentences displayed on a screen, which changed every minute. Randomly, they were asked to choose a song and then, after a countdown, stand up and sing it in front of the experimenter and other participants. After the stress induction, participants were randomly assigned to draw with oil pastels or on the tablet for 45 min. See Figure 1 for a flow chart of the procedure.

### 2.3. Group Sing-a-Song Stress Test (SSST-G)

SSST is a validated stress induction protocol approved for human research. It is easy to administer, and it minimizes variability in sensory input and body movement. In the stress interval, the participant had to prepare and sing a song aloud in front of two people (Brouwer & Hogervorst, 2014). A preliminary study confirmed the reliability of the group format (Czamanski-Cohen & Galili, 2020). In the present study, 10 participants sat in a semicircle and read neutral sentences on a screen that changed every minute. At a random point, they were instructed to prepare a song and then sing it aloud.

### 2.4. Ethics and Confidentiality

Participants signed an informed consent form and were informed they could withdraw from the study at any time without penalty. The study was approved by the ethics committee of the Faculty of Social Welfare and Health Sciences at the University of Haifa (approval number 438-17).

### 2.5. Measures

A demographic questionnaire assessed occupation, age, marital status, previous art experience, tablet ownership, and prior use of digital drawing apps. It also included screening questions related to cortisol measurement, such as alcohol, caffeine, nicotine, medication use, physical activity, and oral health in the 12 h before participation.

State anxiety was measured with the *State Anxiety Questionnaire (STPI)*, a 10-item scale measuring on a 1 to 5 Likert scale. Higher scores reflected higher anxiety. The Hebrew version has strong reliability and internal consistency (Zeidner & Ben-Zur, 1989), and in this study, the Cronbach’s alpha was 0.85.

Participants’ experiences during artmaking were measured with the *Art-Based Intervention Questionnaire (ABI)*, a 41-item self-report measure. It includes scales such as general experience, product evaluation, playfulness, pleasantness, difficulty, and competence. Reliability in this study ranged from acceptable to high (Snir & Regev, 2013).

Emotional valence, arousal, and dominance were measured with the *Self Assessment Manikin (SAM)*, a nonverbal pictorial evaluation tool rated on a 9-point scale. It has demonstrated good reliability and validity in measuring emotional responses (Bradley & Lang, 1994).

The nine dimensions of flow were measured using the *Flow State Scale (FSS)*, a 36-item questionnaire with responses rated from 1 to 5 (Jackson & Marsh, 1996). Internal consistency in this study ranged from Cronbach’s alpha 0.42 to 0.91, depending on the subscale.

### 2.6. Cortisol Measurement

Two saliva samples were collected with SalivaBio oral swabs: one before the stress induction and one 45 min after artmaking. Samples were processed using Salimetrics cortisol Enzyme Immunoassay Kit, which is an ELISA kit (R&D Systems), and analyzed according to standard protocols (Czamanski-Cohen et al., 2016).

### 2.7. Statistical Analysis

Statistical tests were performed using SPSS version 25 (IBM, Chicago, IL, USA). Analyses included repeated-measures ANOVA and paired *t*-tests to examine differences between and within groups for cortisol, anxiety, flow, and emotional state.

## 3. Results

After examining the demographic characteristics (see Table 1), it was found that 21 participants did not meet the conditions required for valid cortisol measurement. These participants were excluded for the following reasons: 10 participants consumed caffeine within 12 h before the experiment; 3 participants took medication and consumed caffeine; 1 participant consumed caffeine and performed intense exercise; and 7 participants took medication within 12 h before the experiment. The means and standard deviations of cortisol levels at baseline and after drawing for both groups are presented in Table 2. A *t*-test for independent samples showed no significant difference in baseline cortisol levels between the two groups. t(23) = −1.28, NS. A paired samples *t*-test examined whether cortisol decreased after artmaking. For the oil pastel group (n = 14), there was no significant change between the before-drawing mean of 2.56 (±3.59) ng/mL as compared to the after-drawing mean of 3.32 (±3.56) ng/mL; t(13) = −0.53, NS. For the tablet group (n = 11), there was no significant change between the before-drawing mean of 4.45 (±3.76) ng/mL as compared to the after-drawing mean of 2.95 (±3.47) ng/mL; t(10) = 2.02, NS.

Repeated-measures ANOVA with time as a within-subjects factor (baseline vs. post) and group as a between-subjects factor did not yield significant effects for time, group, or interaction. Time F(1, 23) = 0.17, NS; Group F(1, 23) = 0.42, NS; Interaction F(1, 23) = 1.67, NS.

The second part of the hypothesis predicted a decrease in state anxiety. Means and standard deviations for both groups at both time points are shown in Table 3. As hypothesized, the oil pastel group showed a significant decrease in anxiety from the before-drawing mean (SD) of 22.32 (±7.34) to the after-drawing mean (SD) of 16.16 (±5.29), t(24) = 3.65, *p* = 0.001. Similarly, the tablet group showed significant decreases in from the before-drawing mean (SD) 22.19 (±5.59) to the after-drawing mean (SD) 16.85 (±5.77); t(20) = 3.65, *p* < 0.01 A repeated-measures ANOVA showed a significant effect of time F(1, 44) = 25.47, *p* < 0.001, with no significant group effect F(1, 44) = 0.04, NS, and no significant interaction F(1, 44) = 0.13, NS. This means that both groups showed a significant reduction in anxiety after drawing, with no difference between materials.

To examine differences in emotional state between the two groups, emotional response scores were analyzed using repeated-measures ANOVA. The within-subjects factor was time of measurement (before drawing and after drawing). The between-subjects factor was group (oil pastels or tablet). The analysis was conducted for each subscale of the task: valence, arousal, and dominance. The means and standard deviations appear in Table 3. The repeated-measures ANOVA examined whether emotional response changed from before to after drawing in each group, and whether the amount of change differed between the two groups. Both groups showed emotional changes from before to after the drawing activity, but no significant differences were found between the oil pastel group and the tablet group in the magnitude of emotional change. This applies to all three measured emotional dimensions: valence, arousal, and dominance (see Table 4).

To examine whether there were differences between the two groups in flow experience during the drawing task, flow scores were analyzed using independent samples *t*-tests. The flow measures comprised nine subscales; each subscale was examined separately (see Table 4). The analyses showed no significant differences between the oil pastel group and the tablet group in any of the flow subscales. The two groups reported similar levels of flow experience during the drawing activity. This pattern was consistent across the overall flow score and across all nine dimensions of the Flow State Scale. This means that the subjective experience of immersion, concentration, clarity of goals, loss of self-consciousness, sense of control, and the other dimensions that make up flow did not differ between creating with traditional materials and creating on a tablet. Similarly, the artmaking experience (pleasantness, sense of competence, difficulty executing, playfulness, attitude toward the product, response during the process, and general art-based assessment) did not differ between the oil pastel and tablet groups (see Table 4).

The results show that both groups experienced a significant decrease in state anxiety after the drawing activity, with no differences between drawing on a tablet and drawing with oil pastels. There was no significant decrease in cortisol levels in either group following the drawing. Emotional response measures showed that both groups exhibited changes before and after drawing, with no significant differences between groups. Flow and artmaking experience did not differ between the two groups. In summary, the findings across all physiological and emotional measures indicate that drawing, regardless of whether on a tablet or with traditional materials, yielded similar patterns of stress and emotional experience.

## 4. Discussion

The present study investigated whether drawing with traditional art materials or on a digital tablet differentially influenced psychological and physiological stress responses following a group stress induction. Contrary to the initial hypotheses, both modalities produced comparable reductions in state anxiety and similar emotional responses, with no significant differences in cortisol levels or flow experience. These findings provide an opportunity to interpret the mechanisms of artmaking through the lens of the ETC and to consider how different media may engage distinct levels of processing.

The ETC proposes that art media vary in the degree to which they activate sensory, affective, perceptual, cognitive, and integrative processes (Kagin & Lusebrink, 1978; Lusebrink, 2010). Traditional materials such as oil pastels are typically associated with the kinesthetic–sensory and perceptual–affective levels because they offer tactile feedback, variable resistance, and direct physical interaction (Hinz, 2009). Digital drawing tools, by contrast, are often conceptualized as leaning toward the perceptual–cognitive levels due to reduced tactile sensation and the presence of interface-mediated controls (Moon, 2010). Based on this framework, one might expect traditional materials to elicit stronger affective engagement and deeper sensory immersion, potentially leading to greater emotional expression or stress reduction.

However, the current findings suggest that the therapeutic benefits of artmaking may not be strictly determined by the material’s position on the ETC, but rather by the broader process of creative engagement. Both digital and traditional drawing appear capable of activating a combination of sensory, perceptual, and affective processes sufficient to support emotional regulation. This aligns with research showing that creative activity itself, regardless of medium, can reduce stress and promote well-being (Kaimal et al., 2016).

One explanation consistent with contemporary ETC theory is that media do not rigidly dictate the level of processing; instead, the user’s interaction style shapes which ETC level becomes dominant (Lusebrink & Hinz, 2016). Participants drawing on the tablet may have engaged with the medium in a fluid, exploratory manner that activated sensory–affective processes despite the lack of tactile feedback. Conversely, participants using oil pastels may have worked in a more structured or representational way, shifting their engagement toward perceptual or cognitive levels. This flexibility reflects ETC’s emphasis on the dynamic interplay between media properties, individual preferences, and contextual factors (Lusebrink & Hinz, 2016). Another potential explanation is that the technology embedded in digital media today provides a sensory experience similar to that of traditional materials (Sion et al., 2023).

The absence of differences in flow experience further supports the idea that both modalities can facilitate a creative, integrative state. Flow involves a merging of action and awareness, reduced self-consciousness, and intrinsic motivation (Csikszentmihalyi, 1990). Prior research indicates that flow can emerge across a wide range of creative tasks and media (Chilton, 2013), suggesting that the immersive qualities of the drawing task itself may have been sufficient to support integrative processing in both groups.

The lack of cortisol reduction in either group, despite decreases in self-reported stress, highlights another important ETC-related distinction: psychological and physiological regulation may operate through different pathways. This contradicts earlier findings showing decreased cortisol after artmaking, such as the reduction observed after 45 min of free creation in Kaimal et al. (2016). In the present study, the cortisol rise following the stress induction might not have been fully captured because measuring cortisol increase would have required interrupting the drawing task. While the creative process may have effectively supported emotional regulation at the affective and cognitive levels, the physiological stress response may require either a longer recovery period or a different type of sensory–kinesthetic engagement to produce measurable hormonal changes. Some ETC theorists argue that kinesthetic or highly sensory activities may be more effective for down-regulating physiological arousal (Hinz, 2009), suggesting that future studies could compare more tactile- or movement-based art tasks with digital modalities.

Overall, the findings contribute to ETC theory by demonstrating that digital artmaking can be just as effective as traditional materials in supporting emotional regulation, challenging assumptions that digital tools inherently limit sensory or affective engagement. Instead, the results suggest that the ETC should be understood as a dynamic system in which media characteristics, user interaction, and task structure jointly shape therapeutic outcomes. These results underscore the importance of considering individual differences and contextual factors when applying the ETC to clinical or research settings.

The study has several limitations that should be taken into consideration when interpreting its findings. One limitation concerns the cortisol measurements. A large number of participants did not meet the required conditions for accurate cortisol assessment and were therefore excluded from the analysis. Another limitation relates to the timing of cortisol sampling. To properly detect an increase in cortisol following the stress induction, it would have been necessary to interrupt the drawing activity and collect an additional sample approximately 20–25 min after the induction. Because the study aimed to avoid interrupting the creative process, this intermediate sample was not collected. As a result, it is unclear whether cortisol levels rose following the stress induction or whether the absence of a decrease after drawing was due to this missing measurement. It is also possible that the stress induction increased cortisol levels to a point where 45 min of artmaking was insufficient to restore the baseline. The specific timing of the second sample may have missed the physiological trough of the stress recovery curve. Prior research indicates that sex differences exist in cortisol patterns, and therefore, the results cannot be generalized to men. The study also focused on a relatively young and healthy population, most of whom were students, which may limit the applicability of the findings to clinical populations or to older adults. Finally, although the study compared two types of materials, it did not include a no-art control condition. Furthermore, the digital app was only compared to oil pastels. We do not know whether we would get the same findings when comparing watercolors to the digital app. In our case, we limited participants to using only the digital app’s oil pastel option for comparison. However, digital apps usually allow for a wider range of materials; we do not know how this selection choice, compared to the oil pastel option, might have affected the results. In addition, it cannot be conclusively determined whether the reductions in anxiety and changes in physiological indicators were caused specifically by drawing or by other factors such as sitting quietly, focusing attention, or interacting with the environment. The study’s findings highlight several directions for future research to deepen understanding of emotional and physiological responses during artmaking with traditional materials and digital tools.

The therapeutic relevance of tablet-based drawing may be especially meaningful for populations with sensory sensitivities or mobility limitations. Future studies may also benefit from including a no-art control condition. This would help clarify whether the observed reduction in anxiety and changes in physiological indicators result specifically from artmaking or could be attributed to other factors such as rest, focused attention, or group presence. Finally, qualitative methods such as interviews, observations, and analyses of artwork could deepen understanding of participants’ subjective experiences. These methods could reveal nuances in how individuals perceive traditional versus digital materials and what unique therapeutic qualities each medium may offer.

Overall, the results of this study suggest that drawing on a tablet may serve as a valid alternative or addition to traditional art materials in reducing psychological stress and supporting the emotional experience of artmaking. The findings contribute to expanding the understanding of digital tools in art therapy and highlight the potential therapeutic value of both modalities. In addition, our results support the call for the integration of digital tools into a theory that promotes sensory, spatial, and emotional processes using a mix of traditional and digital tools, as proposed by the Phygital Art Therapy model (Yoon et al., 2025). This model views art therapy as one in which the art therapist controls the therapeutic environment, engaging with traditional and digital media to reconfigure sensory experience, symbolic expression, and relational dynamics. Perhaps it is time to integrate digital tools in the art room rather than viewing them as separate modes of engagement. Further studies should examine how digital tools can be integrated into the ETC.

## Figures and Tables

**Table 1:** Demographics.

			Oil Pastels	Tablet
		*N* (%)	*N* (±/%)	*N* (±/%)
Age			23.95 (±7.49)	21.92 (±1.98)
Gender	Female	48 (100)	22 (46)	26 (54)
Birth country	Israel	43 (89.6)	21 (96)	22 (85)
	Other	5 (10.4)	1 (4)	4 (15)
Family status	Single	43 (89.6)	19 (86)	24 (92)
	Married	5 (10.4)	3 (14)	2 (8)
Children	Has children	3 (6.3)	3 (14)	0
Education *	High school	48 (47.9)	15 (68)	9 (35)
	BA	24 (50)	7 (32)	16 (62)
	Graduate degree	1 (2.1)	0	1 (3)
Employment	Working	21 (43.8)	10 (46)	11 (42)
	Not Working	27 (56.2)	12 (54)	15 (58)
Religion	Jewish	26 (54.2)	13 (61)	13 (50)
	Muslim	14 (29.2)	6 (27)	8 (31)
	Christian	6 (12.5)	1 (4)	5 (19)
	Druze	2 (4.1)	2 (8)	0
Religiosity	Secular	26 (54.2)	15 (68)	11 (42)
	Traditional	15 (31.3)	4 (18)	11 (42)
	Religious	6 (12.5)	3 (14)	3 (12)
	Other	1 (2)	0	1 (4)
Art Habit	Not	30 (62.5)	13 (59)	17 (65)
	As a Hobby	17 (35.4)	8 (36)	9 (35)
	Professionally	1 (2.1)	1 (5)	0
Owns tablet	Yes	16 (33.3)	7 (32)	9 (35)

* t [46] = −2.5; *p* = 0.015.

**Table 2:** Mean stress and mean cortisol levels in both groups before and after drawing.

Group	Oil Pastel	Tablet
Baseline Stress M (±SD)	22.32 (±7.43)	22.19 (±5.59)
After-drawing Stress M (±SD)	16.16 (±5.29)	16.85 (±5.77)
T(df)	3.65 (24) **	3.65 (20) **
Baseline Cortisol M (±SD)	2.56 (±3.59)	4.45 (±3.76)
After drawing M (±SD)	3.32 (±3.56)	2.95 (±3.47)
T(df)	−0.54 (13)	2.02 (10)

** *p* < 0.01.

**Table 3:** Emotional response before and after drawing.

	Group	Baseline M (±SD)	After Drawing M (±SD)	T (df)
Valence	Oil Pastel	3.62 (±1.78)	2.56 (±1.59)	2.82 (23) **
	Tablet	3.77 (±1.81)	2.47 (±1.23)	3.08 (21) **
Arousal	Oil Pastel	5.2 (±2.02)	7.06 (±1.91)	−3.21 (23) **
	Tablet	5.77 (±2.36)	7.22 (±1.90)	−2.66 (21) *
Dominance	Oil Pastel	5.25 (±1.56)	6.06 (±1.89)	−1.41 (23)
	Tablet	6.09 (±2.20)	5.59 (±1.99)	1.01 (21)

* *p* < 0.05; ** *p* < 0.01.

**Table 4:** Emotional state, state anxiety, flow, and artmaking experience in both groups.

		M (±SD)	M (±SD)	T (df)
Emotional state	Valence	2.7 (±1.7)	2.47 (±1.23)	0.50 (45)
	Arousal	6.82 (±2.23)	7.22 (±1.90)	−0.66 (45)
	Dominance	5.98 (±1.9)	5.59 (±1.99)	0.68 (45)
State anxiety		16.50 (±5.47)	16.59 (±5.77)	−0.05 (46)
Flow	Balance between skills and challenge	3.34 (±0.90)	3.64 (±0.873)	−1.18 (45)
	Merging of awareness and action	3.48 (±0.9)	3.48 (±1.183)	−0.02 (45)
	Setting clear goals	3.15 (±0.98)	3.22 (±1.27)	−0.23 (45)
	Unambiguous feedback	2.98 (±1.08)	3.39 (±1.02)	−1.35 (45)
	Concentration on the task	3.72 (±0.76)	4.31 (±1.90)	−1.44 (45)
	Sense of control	3.81 (±0.96)	4.01 (±0.94)	−0.73 (45)
	Loss of public self-consciousness	3.96 (±0.88)	4.07 (±0.87)	−0.46 (45)
	Altered sense of time	3.27 (±0.87)	3.25 (±1.11)	0.06 (45)
	Autotelic experience	3.83 (±0.76)	3.97 (±0.79)	−0.63 (45)
	Overall flow	3.50 (±0.69)	3.70 (±0.74)	−0.95 (45)
Artmaking experience	Pleasantness	5.02 (±1.24)	5.26 (±1.24)	−0.68 (46)
	Sense of competence	4.93 (±1.45)	5.02 (±1.18)	−0.22 (46)
	Difficulty executing	2.27 (±1.21)	2.12 (±0.99)	0.47
	Playfulness	5.48 (±1.43)	5.75 (±1.0)	−0.73
	Attitude to product	4.76 (±1.50)	5.20 (±1.13)	−1.13
	Response during the process	5.12 (±1.09)	5.30 (±0.96)	−0.61
	General art-based assessment	5.04 (±1.13)	5.28 (±0.93)	−0.79

## Data Availability

Data is unavailable due to ethical restrictions. The raw data supporting the conclusions of this article will be made available by the authors on request.

## Abbreviations

The following abbreviations are used in this manuscript:

SOC	Sense of Coherence
ETC	Expressive Therapies Continuum
SSST-G	Group Sing-a-Song Stress Test
